# Ultrasound-Assisted Enzymatic Extraction of the Active Components from *Acanthopanax sessiliflorus* Stem and Bioactivity Comparison with *Acanthopanax senticosus*

**DOI:** 10.3390/molecules30020397

**Published:** 2025-01-18

**Authors:** Qiaomu You, Yanjun Ren, Jiaotong Li, Guangnian Zeng, Xiongfei Luo, Chunying Zheng, Zhonghua Tang

**Affiliations:** 1 Key Laboratory of Forest Plant Ecology of Ministry of Education, Northeast Forestry University, Hexing Road 26, Harbin 150040, China; yjcyqm@126.com (Q.Y.); 609878210@nefu.edu.cn (Y.R.); ljiaot@nefu.edu.cn (J.L.); zengguangnian@163.com (G.Z.); tangzh@nefu.edu.cn (Z.T.); 2 Engineering Research Center of Agricultural Microbiology Technology, Ministry of Education & Heilongjiang Provincial Key Laboratory of Plant Genetic Engineering and Biological Fermentation Engineering for Cold Region & Key Laboratory of Microbiology, College of Heilongiang Province & School of Life Sciences, Heilongiiang University, Harbin 150080, China

**Keywords:** *Acanthopanax sessiliflorus*, ultrasound-assisted enzymatic extraction, antioxidant, antibacterial, utilization of plant resources

## Abstract

*Acanthopanax senticosus* (ASC) contains a variety of bioactive compounds and serves as an important traditional Chinese medicinal resource. However, its prolonged growth cycle and reliance on wild populations limit its practical use. To explore the potential of *Acanthopanax sessiliflorus* (ASF) as an alternative, this study focused on optimizing the extraction process and assessing the bioactivity of stem extracts. The Analytic Hierarchy Process-Entropy Weight Method (AHP-EWM) was applied to comprehensively score five key active compounds in ASF stems, establishing a benchmark for evaluating extraction efficiency. Ultrasound-assisted enzymatic extraction (UAEE) was employed, and orthogonal and response surface experiments were conducted to refine the extraction parameters. The optimal conditions identified were an ultrasound temperature of 59 °C, a duration of 57 min, an ethanol concentration of 61%, and a liquid-to-material ratio of 39:1, resulting in an AHP-EWM composite score of 94.76. Comparative analysis of antibacterial and antioxidant activities revealed that ASC extracts exhibited superior antibacterial effects, while ASF extracts demonstrated enhanced antioxidant properties. These findings support the partial replacement of ASC with ASF, contributing to the conservation of wild resources and expanding the application of ASF in medicinal development.

## 1. Introduction

The genus *Acanthopanax* comprises approximately 37 species worldwide, with China hosting 26 species and 18 varieties [1]. *Acanthopanax senticosus* (ASC), a traditional Chinese medicinal plant, is known for its diverse bioactive compounds, including flavonoids, eleutherosides, and polysaccharides. Among these, eleutheroside E and eleutheroside B are the primary active components, exhibiting significant health-promoting effects such as antioxidant, anti-inflammatory, immunomodulatory, antifatigue, and cardiovascular protective activities [2,3]. Additionally, the polysaccharides and flavonoids present in ASC display potent antioxidant properties, serving as natural agents capable of scavenging reactive oxygen species and hydroxyl radicals, thus mitigating aging, accelerating recovery, and enhancing immunity.

Traditionally, the roots, stems, and leaves of ASC are utilized for medicinal purposes, mainly for immune modulation and antifatigue benefits [4]. However, the increasing market demand and ASC’s lengthy growth cycle have led to resource shortages and the depletion of wild populations. Consequently, there is an urgent need to develop efficient cultivation strategies or identify alternative sources with similar bioactive profiles to ensure the sustainable utilization of these valuable plant resources.

*Acanthopanax sessiliflorus* (ASF) emerges as a promising alternative, given its phylogenetic similarity to ASC and common presence in Northeast China. Research indicates that ASF stems contain bioactive compounds analogous to those in ASC [5], exhibiting analgesic, anti-inflammatory, and antioxidant properties [6,7]. Moreover, ASF offers distinct advantages over ASC, such as a shorter growth cycle, reduced cost, and greater accessibility [8]. Despite these potential benefits, the optimization of extraction methods for ASF’s active components remains underexplored, necessitating efforts to enhance extraction efficiency and evaluate the bioactivity of the derived extracts.

Conventional extraction techniques for bioactive components from ASF, such as Soxhlet extraction and heat reflux [9], present significant limitations, including prolonged extraction times, elevated temperatures, low yields, and potential degradation of sensitive compounds [10]. To address these challenges, advanced extraction methods like ultrasound-assisted extraction (UAE), enzyme-assisted extraction (EAE), and microwave-assisted extraction (MAE) have been developed, offering higher efficiency, environmental sustainability, and milder conditions [11]. Moreover, combining two advanced extraction techniques, such as ultrasound-assisted enzymatic extraction (UAEE) and microwave-assisted extraction (MAE), can greatly enhance extraction efficiency [12]. Theoretically, UAEE utilizes the synergistic effects of ultrasound and enzymatic action to thoroughly disrupt plant cell walls, thereby facilitating the release of active compounds and significantly improving extraction yield [13].

The Analytic Hierarchy Process-Entropy Weight Method (AHP-EWM) is a comprehensive evaluation approach that combines subjective judgment with objective data. The Analytic Hierarchy Process (AHP) establishes a multilevel index system and determines weights based on expert scoring, while the Entropy Weight Method (EWM) objectively assigns weights according to the variability of index data. By integrating these two methods, AHP-EWM effectively overcomes the limitations of using a single approach, ensuring more comprehensive and scientifically robust evaluation results.

This study employs the AHP-EWM to score five key bioactive components—eleutheroside B, eleutheroside E, chlorogenic acid, hyperoside, and isofraxidin—within ASF stems, establishing a benchmark for evaluating extraction efficiency. The Box-Behnken Design (BBD) is applied to optimize the UAEE process for these active components. Subsequent bioactivity assessments of extracts obtained from ASF and ASC stems under optimized conditions provide insights into the potential of ASF as a resource for medicinal development while also emphasizing the need to conserve wild ASC populations. Our findings have the potential to inform both practical applications and future research directions in the sustainable development of medicinal plants.

## 2. Results and Discussion

### 2.1. Determination of the Optimal Compound Enzyme Ratio

#### 2.1.1. Single-Factor Experiment Results for Enzymes

**Figure 1:**
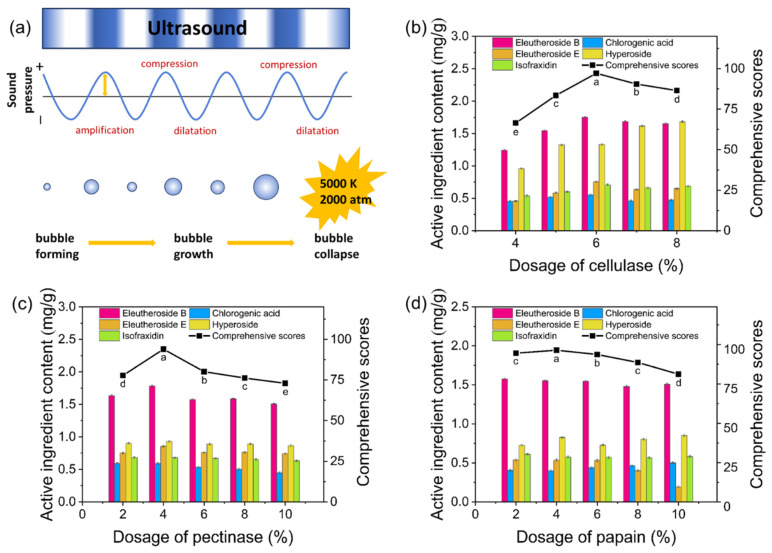
Cavitation effect of ultrasound and results of single-factor experiments for the composite enzyme. (**a**) Illustration of the cavitation effect, (**b**) effect of cellulase dosage, (**c**) effect of pectinase dosage, (**d**) effect of papain dosage. Note: According to ANOVA, different letters (a, b, c, d, e) indicate significant differences (*p* < 0.05), while the same letter denotes no significant difference.

Ultrasound-assisted extraction enhances efficiency through the cavitation effect, wherein ultrasound waves cause periodic compression and expansion of medium molecules as they propagate, leading to bubble formation and subsequent collapse in the liquid medium (Figure 1a) [14]. Plant cell walls, composed of cellulose, pectin, and proteins, can be degraded by cellulase, pectinase, and papain, respectively. This enzymatic action disrupts the cell structure, facilitating the release of active components [15]. Additionally, the synergistic effect of these three enzymes significantly improves the extraction efficiency and quality of active components while reducing both extraction time and energy consumption. Therefore, the UAEE method was employed to extract active components from ASF.

Cellulase, pectinase, and papain were selected for the extraction experiments. The effect of cellulase dosage on the extraction efficiency and its comprehensive score is shown in Figure 1b. As the major bioactive constituents in ASF, the contents of eleutheroside E and eleutheroside B exhibited trends corresponding to changes in cellulase dosage. The comprehensive score increased initially and then declined, reaching a peak at a cellulase dosage of 6% (Figure 1b). Similar patterns were observed with pectinase and papain, where the comprehensive score maximized at dosages of 4% for both enzymes (Figure 1c and Figure 1d, respectively). These findings indicate that enzyme dosage significantly influences extraction efficiency, highlighting the need to optimize the enzyme ratios for mixed-enzyme extractions.

#### 2.1.2. Orthogonal Result

Orthogonal experiments were conducted to determine the optimal enzyme ratios, as shown in Table 1 and Table 2. Analysis of the results from nine experimental runs indicated variations in extraction efficiency across different enzyme combinations, with cellulase exerting the greatest influence, followed by papain and pectinase. This priority ranking was consistent with the findings from the single-factor experiments. The optimal enzyme combination for maximizing active component extraction was identified as cellulase:pectinase:papain = 7%:5%:5% (Table 1 and Table 2). ANOVA results for the orthogonal test (Table 3) indicated statistically significant differences, with F values of 1106.07, 19.88, and 38.93 and corresponding *p*-values of 0.001, 0.048, and 0.025, respectively.

### 2.2. Study on the Optimization of Active Component Extraction from ASF Stems Using Response Surface Methodology

#### 2.2.1. Analysis of Single-Factor Test Results

**Figure 2:**
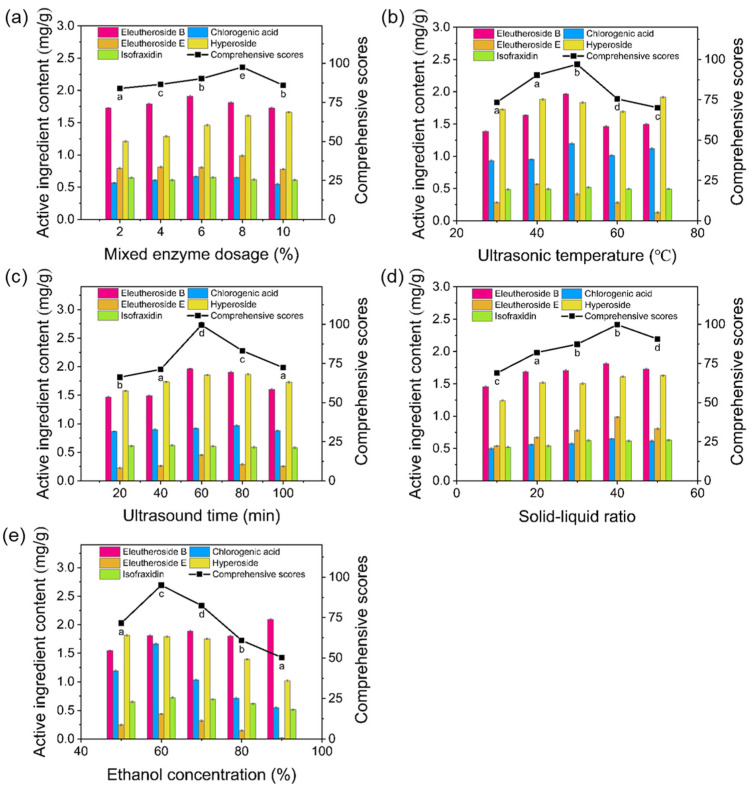
Effects of various extraction parameters on the comprehensive score. (**a**) Mixed enzyme dosage, (**b**) ultrasonic temperature, (**c**) ultrasound time, (**d**) solid-to-liquid ratio, (**e**) ethanol concentration. Note: According to ANOVA, different letters (a, b, c, d, e) indicate significant differences (*p* < 0.05), while the same letter denotes no significant difference.

The effects of mixed enzyme dosage, ultrasonic temperature, ultrasonic time, ethanol concentration, and solid-to-liquid ratio on the comprehensive score were further evaluated (Figure 2). The results showed that increasing the mixed enzyme dosage initially enhanced the comprehensive score, reaching a peak at 8% (Figure 2a). This optimal dosage likely reflects a balance where the enzyme mix effectively disrupts the cell walls, maximizing the release of active components. However, beyond this concentration, mutual inhibition among the enzymes may occur, reducing overall enzyme activity and thereby affecting extraction efficiency.

As illustrated in Figure 2b, the optimal ultrasonic temperature for maximizing the comprehensive score was found to be 50 °C. This may be due to the enhanced synergistic effect of the enzyme mix at this temperature, while higher temperatures likely cause enzyme deactivation, leading to a decline in extraction efficiency [16]. Figure 2c demonstrates that the optimal ethanol concentration for extracting active components from ASF stems was 60%. Higher ethanol concentrations appeared to inhibit enzyme activity, thereby decreasing the efficiency of cell wall degradation and reducing the release of active components [17]. Additionally, the influence of the solid-to-liquid ratio and ultrasonic time was assessed. The comprehensive score peaked at a solid-to-liquid ratio of 40:1 and an ultrasonic time of 60 min.

Based on these findings, the ultrasonic temperature, ultrasonic time, ethanol concentration, and solid-to-liquid ratio were selected for subsequent response surface experiments to further optimize the extraction process.

#### 2.2.2. Model Fitting

The four parameters were optimized within the following ranges: an ultrasonic temperature from 30 °C to 70 °C, an ultrasound time from 20 to 100 min, a solid-to-liquid ratio from 10:1 to 50:1, and an ethanol concentration from 30% to 70%, as indicated by the results of the single-factor experiments. A total of 29 experiments were conducted to finetune these operating parameters, with the comprehensive scores for each combination of independent factors shown in Table 4. The relationships between the UAEE parameters and the comprehensive score were modeled using a second-order quadratic equation, incorporating coded variables to represent the independent factors Equation (1):

(1) $$Comprehensive\;score=-554.648-8.903A+3.256B+7.097C+5.697D-\;0.010AB+0.016AC+0.001AD-0.002BC+0.009BD+0.008CD-0.089A^{2}-\;0.025B^{2}-0.068C^{2}-0.086D^{2}$$

In this equation, A represents the ultrasonic temperature, B represents the ultrasonic time, C represents the ethanol concentration, and D represents the solid-to-liquid ratio).

The ANOVA results for the response surface analysis were statistically evaluated, assessing the significance of each coefficient through F-tests and *p*-values (Table 5). The model showed a *p*-value of less than 0.0001, indicating a robust fit between the comprehensive score and the independent parameters, thus effectively explaining their correlation. The nonsignificant lack of fit (*p* > 0.05) suggested that the model’s residuals did not significantly deviate from random error, confirming a good fit to the experimental data and successful construction of the response surface [18]. The coefficient of variation (C.V.%) was below 10%, reflecting high consistency and reliability in the experimental results, with precise measurements and accurate model predictions [19]. The regression analysis of the comprehensive score showed a coefficient of determination (R^2^) of 0.9992 and an adjusted R^2^ of 0.9983, both approaching unities. The small difference between predicted R^2^ and adjusted R^2^ (less than 0.2) confirmed a strong correlation between observed and predicted values, validating the model’s accuracy and suitability for simulating and analyzing the experimental outcomes [20].

The regression models indicated that the independent factors had linear effects on the comprehensive score in the UAEE process. Ultrasonic temperature had the most significant impact (*p* < 0.0001), followed by ultrasound time (*p* < 0.0001), solid-to-liquid ratio (*p* < 0.0001), and ethanol concentration (*p* < 0.001). The quadratic terms (A^2^, B^2^, C^2^, D^2^) and interaction terms (AB, AC, BD, CD) were highly significant (*p* < 0.0001), while BC was significant at the *p* < 0.01 level (Table 5). The interaction of AD, however, did not show a significant effect on the comprehensive score. The F-values for each term highlighted the relative influence on the comprehensive score, with higher F-values indicating greater impact. Ultrasonic temperature (factor A) had the strongest effect on the comprehensive score of ASF stems. The influence of individual factors followed the order: ultrasonic temperature > solid-to-liquid ratio > ultrasound time > ethanol concentration [21]. This trend can be attributed to the optimal temperature for enzyme activity being around 50 °C, where temperatures that deviate significantly lower or higher adversely affect enzyme activity, thus impacting the efficiency of active component extraction from ASF stems.

#### 2.2.3. Analysis of Response Surface Curves

**Figure 3:**
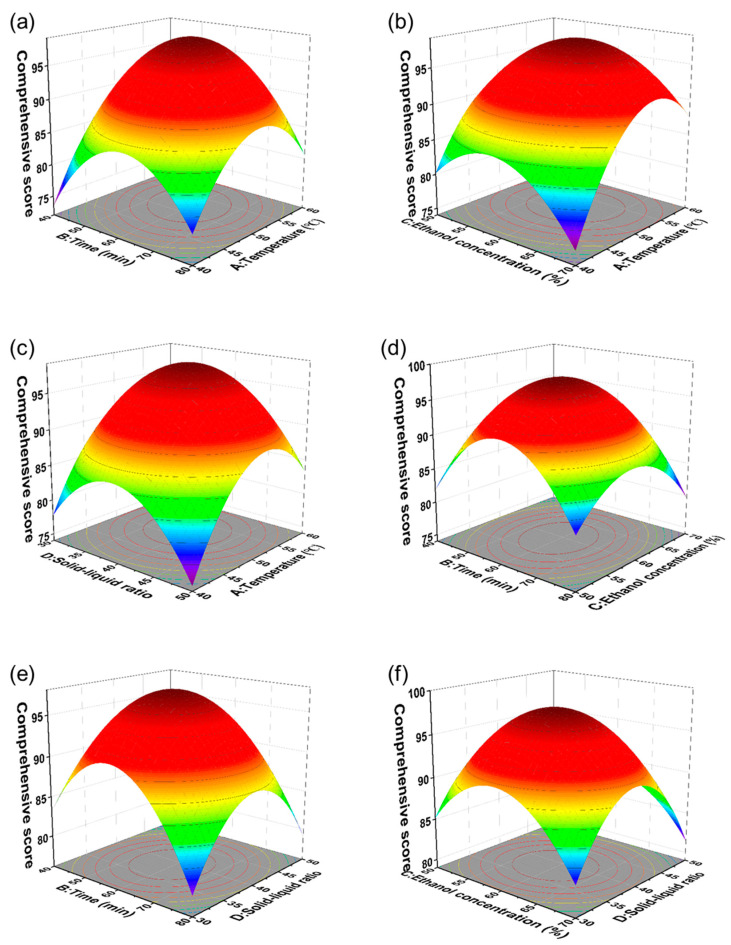
Response surface plots illustrating the effects of independent variables on the comprehensive score. (**a**) ultrasonic temperature vs. ultrasound time, (**b**) ultrasonic temperature vs. ethanol concentration, (**c**) ultrasonic temperature vs. solid-to-liquid ratio, (**d**) ultrasound time vs. ethanol concentration, (**e**) ultrasound time vs. solid-to-liquid ratio, (**f**) ethanol concentration vs. solid-to-liquid ratio.

The combined effects of the two operational parameters were visualized through three-dimensional response surface curves derived from the regression equations (Figure 3). These curves depicted the responses, trial levels, and interactions for each of the four investigated variables [22]. The response surfaces across the different factor interactions exhibited steep, bell-shaped contours, while the corresponding contour plots in the lower projections appeared tight and elliptical. This pattern indicated significant factor interactions, corroborating the ANOVA findings in Table 5 and confirming the reliability of the response surface model.

The comprehensive score demonstrated a clear trend, initially increasing and then decreasing as the ultrasonic temperature rose from 40 to 60 °C, the ultrasound time extended from 40 to 80 min, the ethanol concentration increased from 50% to 70%, and the solid-to-liquid ratio expanded from 30:1 to 50:1 (Figure 3a–f). The peak comprehensive scores consistently appeared near the center of the response surfaces, suggesting that the optimal extraction conditions for the active components from ASF stems were close to the midpoint of each variable. Using Design Expert 13 for optimization, the ideal extraction conditions were identified as follows: an ultrasonic temperature of 58.94 °C, an ultrasonic time of 57.30 min, an ethanol concentration of 60.73%, and a solid-to-liquid ratio of 39.16:1. Under these optimal conditions, the comprehensive AHP-EWM score for the active components of ASF stems reached 94.76.

### 2.3. Model Validation

Based on the results and practical considerations for production, the optimal extraction parameters were adjusted to an ultrasonic temperature of 59 °C, an ultrasound time of 57 min, an ethanol concentration of 61%, and a solid-to-liquid ratio of 39:1. Validation experiments, repeated three times, yielded a final comprehensive AHP-EWM score of 95.24 ± 0.12. This score differed by less than 5% from the predicted value, confirming the model’s accuracy and effectiveness in optimizing the process. Under these conditions, the measured concentrations of active components were eleutheroside B at 1.52 mg/mL, chlorogenic acid at 0.60 mg/mL, eleutheroside E at 1.19 mg/mL, isofraxidin at 1.61 mg/mL, and hyperoside at 0.27 mg/mL.

### 2.4. Comparison of UAEE and UAE

To demonstrate the benefits of UAEE, a comparison was made with UAE alone. As shown in Figure S1, the concentrations of eleutheroside B, chlorogenic acid, eleutheroside E, isofraxidin, and hyperoside increased from 1.09 to 1.52, 0.48 to 0.60, 0.57 to 1.19, 1.27 to 1.61, and 0.24 to 0.27 mg/g, respectively, using UAEE. This represents an approximately 30% improvement in extraction efficiency compared to UAE, providing robust scientific evidence for the broader adoption of UAEE for ASF.

### 2.5. Evaluation of Active Components in ASF and ASC

Following the optimization via response surface methodology, UAEE was applied to both ASF and ASC stems, with results shown in Figure S2. The content of eleutheroside E and chlorogenic acid was significantly higher in ASC stem extracts compared to ASF, while eleutheroside B and hyperoside levels were lower in ASC stems than in ASF. These findings align with previous research by Jia et al. [4], which indicated that ASC stems naturally have higher levels of eleutheroside E and chlorogenic acid, whereas hyperoside is more prevalent in ASF stems. Further liquid chromatography analysis confirmed the absence of detectable levels of isofraxidin in ASC stems, indicating that its concentration was below the quantifiable range.

The antibacterial and antioxidant activities of ASF and ASC stem extracts were assessed. As shown in Figure S3 and Table S1, both extracts demonstrated antibacterial properties, with ASC stem extracts exhibiting stronger antibacterial effects overall. Both extracts displayed more potent antifungal activity, likely due to differences in the cell wall structures of bacteria and fungi and the resistance mechanisms of the strains. Specifically, *Candida albicans* was more susceptible to the active components in the extracts, while *Pseudomonas aeruginosa*, *Escherichia coli*, and *Bacillus subtilis* exhibited greater resistance.

**Figure 4:**
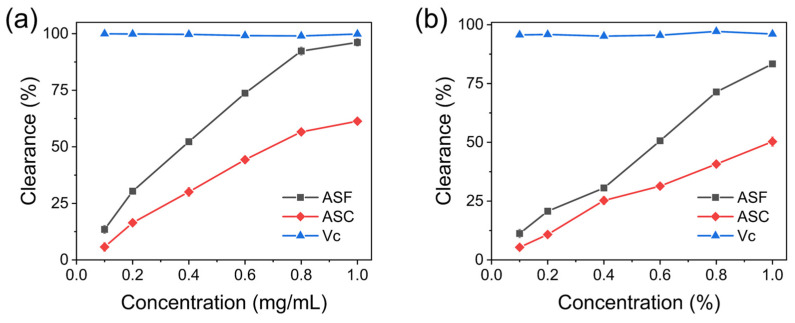
Comparison of antioxidant activity between ASF and ASC stem extracts. (**a**) ABTS radical scavenging activity, (**b**) DPPH radical scavenging activity.

The ABTS and DPPH radical scavenging activities of ASF and ASC stem extracts are presented in Figure 4, showing that both extracts possessed good antioxidant capacity, with scavenging rates increasing with higher extract concentrations. Notably, the antioxidant activity of ASF stem extracts surpassed that of ASC stem extracts, potentially due to higher concentrations of flavonoids, polysaccharides, and other antioxidant compounds in ASF. In the ABTS assay, the antioxidant activity of ASF stem extract at a concentration of 1.0 mg/mL was comparable to that of Vc, suggesting that this concentration achieved an effective level for high antioxidant capacity (Figure 4a) [23].

These findings confirmed that ASF stems contain a range of bioactive components and exhibit antibacterial activity similar to ASC stems. Moreover, the higher antioxidant capacity of ASF stem extracts indicates that ASF could serve as a promising alternative to ASC stems, with potential applications in pharmaceuticals, health supplements, and functional foods.

## 3. Materials and Methods

### 3.1. Sample Preparation

The stems of ASF and ASC were sourced from the cultivation base of the College of Chemical Resources and Utilization at the Northeast Forestry University. The collected stems were dried in an oven (Shanghai Hecheng Instrument Manufacturing Co., Ltd., Shanghai, China) at a stable temperature of 60 °C until a constant weight was achieved. The moisture content of ASF stems is 56.47 ± 0.29%, and the moisture content of ASC is 57.78 ± 14%. Subsequently, the dried samples were finely ground using a grinder (Shanghai Hakun Industrial Co., Ltd., Shanghai, China) and passed through an 80-mesh sieve to ensure uniformity in particle size [21].

### 3.2. Reagents and Chemicals

Anhydrous ethanol, Acetonitrile and Methanol (J&K Scientific Co., Ltd., Beijing, China), Cellulase, Pectinase and Papain (Shanghai yuanye Bio-Technology Co., Ltd., Shanghai, China), Vc, Kanamycin and Agar (Shanghai Acmec Biochemical Technology Co., Ltd., Shanghai, China), Potassium persulfate, DPPH, and ABTS (Merck Sigma-Aldrich Co., Ltd., Darmstadt, Germany), *Pseudomonas aeruginosa*, *Staphylococcus aureus*, *Escherichia coli*, *Bacillus subtilis*, and *Candida albicans* (All provided by the Heilongjiang Provincial Institute of Drug Control and Research, Herbin, China).

### 3.3. HPLC for Detecting Five Compounds

The high-performance liquid chromatography (HPLC) method was adapted from previous reports with necessary modifications [5]. The chromatographic separation was performed using LC-100 HPLC (Shanghai Wufeng Scientific Instrument Co., Ltd., Shanghai, China) equipment with an Agilent Extended-C18 column (4.6 mm × 250 mm, 5 μm). The mobile phase consisted of acetonitrile (Phase A) and 0.1% formic acid solution (Phase B). The elution gradient was programmed as follows: 0–5 min, 8–18% A; 5–15 min, 18–30% A; 15–20 min, 30–40% A; 20–25 min, 40–100% A; and 25–30 min, 100% A. The detection wavelength was set at 220 nm, with a flow rate of 1 mL/min, a column temperature of 30 °C, and an injection volume of 20 μL.

### 3.4. Analytic Hierarchy Process-Entropy Weight Method (AHP-EWM) of Composite Score Establishment

#### 3.4.1. Analytic Hierarchy Process (AHP)

The AHP was employed to establish the weights of the evaluation index system, integrating both qualitative and quantitative assessments. The weight ($r_{i}^{*}$) determination process followed the nine-point scale approach, and the pairwise comparison matrices used for calculations are presented in Table 6 and Table 7 [24].

The m-th root of the product of each row of the comparison matrix $r_{i}$ was calculated using Equation (2).

(2) $$r_{i}=\sqrt[{m}]{\Pi_{j=1}^{m}a_{ij}}$$

In the equation, m represents the number of indicators, and $a_{ij}$ denotes the importance of indicator *i* relative to indicator *j*.

The weight $r_{i}^{*}$ of indicator i was calculated using Equation (3):

(3) $$r_{i}^{*}=\frac{r_{i}}{\sum_{i=1}^{m}r_{i}}$$

The maximum eigenvalue $\lambda_{max}$ was calculated using Equation (4):

(4) $$\lambda_{max}=\frac{1}{m}\sum_{i=1}^{m}\left(\sum_{j=1}^{m}a_{ij}\times \frac{r_{j}^{*}}{r_{i}^{*}}\right)$$

The consistency index *CI* was calculated using Equation (5):

(5) $$CI=\frac{\lambda_{max}-m}{m-1}$$

The consistency ratio *CR* was calculated using Equation (6):

(6) $$CR=\frac{CI}{RI}$$

In this context, *RI* corresponds to the corresponding random index. *CR* serves as a measure of the matrix’s rationality, with a *CR* value below 0.1 indicating acceptable consistency in the assessments; otherwise, the judgments are deemed inconsistent.

Applying the Analytic Hierarchy Process (AHP), the weights for the indicators were calculated as follows: chlorogenic acid, 5.81%; eleutheroside B, 36.72%; eleutheroside E, 36.72%; isofraxidin, 17.62%; and hyperoside, 3.12%. Given n = 5, the corresponding RI was 1.11, resulting in a CR of 0.042, which is below the threshold of 0.1, confirming the reliability of the weight coefficients.

#### 3.4.2. Entropy Weight Method (EWM)

The EWM is a quantitative decision-making technique designed to address multi-objective complex problems. In this method, the weight of each indicator is determined based on the degree of variation across the indicators. A higher degree of variation in an indicator implies that it conveys more information, thereby increasing its significance in the comprehensive evaluation and resulting in a higher assigned weight [25].

The procedure for calculating the weights of the indicators using EWM involved the following steps: data standardization and normalization for each indicator ($P_{ij}^{*}$), computation of the information entropy ($Q_{j}$), and calculation of the index weight ($W_{j}$). These steps ensured an objective assessment of the indicators’ relative importance:

The standardization calculation of indicators is shown in Equation (7):

(7) $$Standardized\;Value=\frac{Measured\;Value-Minimum\;Value}{Maximum\;Value-Minimum\;Value}$$

Normalization $P_{ij}^{*}$ calculation is shown in Equation (8):

(8) $$P_{ij}^{*}=P_{ij}/\sum_{i=1}^{n}P_{ij}$$

Information entropy $Q_{j}$ calculation is shown in Equation (9):

(9) $$Q_{j}=-ln(1/n)\sum_{i=1}^{n}P_{ij}^{*}lnP_{ij}^{*}$$

Index weight $W_{j}$ calculation is shown in Equation (10):

(10) $$W_{j}=\frac{1-Q_{j}}{\sum_{j=1}^{m}\left(1-Q_{j}\right)}$$

In this context, *n* denotes the number of samples.

#### 3.4.3. AHP-EWM

When optimizing multiple components, various indicators can influence the extraction or purification outcomes, necessitating the integration of these factors into a comprehensive score. Determining the priority weights for each component can be challenging. The AHP-EWM method addresses this by combining the content values of multiple components into a unified score, taking into account the influence of each component [25]. The subjective weight coefficients ($r_{i}^{*}$) are derived using the Analytic Hierarchy Process (AHP), while the objective weight coefficients ($w_{i}$) are determined through the EWM [26]. The overall comprehensive weight ($Z_{i}$) is then calculated by integrating both subjective and objective weights, as described in Equation (11):

(11) $$Z_{i}=\frac{r_{i}^{*}w_{i}}{\sum_{i=1}^{m}r_{i}^{*}w_{i}}\left(i=1,2\cdots ,m\right)$$

In this equation, $Z_{i}$ represents the comprehensive weight of the *i*-th indicator calculated using the AHP-EWM method, $r_{i}^{*}$ is the weight of the *i*-th indicator obtained from AHP, and $w_{i}$ is the weight of the i-th indicator derived from EWM.

The comprehensive weights for all indicators, calculated using AHP-EWM, are available in the supplementary data accompanying this study.

#### 3.4.4. Calculation of Comprehensive Score

The comprehensive score *E* was calculated using Equation (12):

(12) $$E=\sum_{i=1}^{m}Z_{i}\times \frac{Q_{i}}{max(Q_{i})}$$

In this equation, $Q_{i}$ represents the content of the i-th indicator.

### 3.5. UAEE

A 0.5 g sample was placed in a 50 mL test tube and mixed with 10 mL of 80% (*v*/*v*) ethanol along with appropriate quantities of enzymes. The mixture was then subjected to ultrasonic extraction using a KQ-500DE ultrasonic instrument (500 W, 40 kHz, Shumei Ultrasonic Instrument Co., Ltd., Kunshan, China) for 60 min at a constant temperature water bath at 40 °C [27].

### 3.6. Compound Enzyme Ratio

#### 3.6.1. Single-Factor Experiment of Enzyme Amounts

The concentrations of cellulase, papain, and pectinase were varied in gradient increments of 2%, 4%, 6%, 8%, and 10%, respectively. The extraction procedure followed the method outlined in Section 2.4, with the pH maintained at 5.5, which is optimal for the activity of all three enzymes [28].

#### 3.6.2. Orthogonal Experiment of the Three Enzymes ($L9{(3}^{4})$)

Based on the outcomes of the single-factor enzyme experiments, the optimal mixed ratio of the three enzymes was determined through an orthogonal experimental design [29]. The details of the orthogonal experiments are presented in Table 1.

### 3.7. Optimization of Active Components Extraction from ASF Stems Using UAEE

The extraction of active components from ASF stems was performed using UAEE, with key parameters including compound enzyme amount, liquid-to-material ratio, ultrasound duration, ultrasound temperature, and ethanol concentration assessed for their impact on extraction yield [30]. The AHP-EWM-derived comprehensive score served as the evaluation metric, guiding the selection of significant factors from single-factor experiments. Response surface experiments conducted via Design Expert 13 further optimized the extraction conditions [31].

#### 3.7.1. Single-Factor Experiments

The extraction procedure followed Section 2.4, with each parameter adjusted across five levels: compound enzyme amount at 2%, 4%, 6%, 8%, and 10%; liquid-to-material ratio at 10, 20, 30, 40, and 50 mL/g; ultrasound duration at 20, 40, 60, 80, and 100 min; ethanol concentration at 50%, 60%, 70%, 80%, and 90%; and ultrasound temperature at 30 °C, 40 °C, 50 °C, 60 °C, and 70 °C [32].

#### 3.7.2. Response Surface Methodology

Response surface methodology was employed to model the relationships between these variables and the extraction outcomes [33]. Based on initial single-factor analyses, a Box-Behnken Design (BBD) with a four-factor, three-level structure was utilized to optimize the UAEE conditions (Table 4) [20]. The independent variables were defined as follows: X1, ultrasound temperature (°C): 40, 50, 60; X2, ultrasound duration (min): 40, 60, 80; X3, ethanol concentration (%): 50, 60, 70; and X4, liquid-to-material ratio (mL): 30, 40, 50. The response variable was Y1 (comprehensive score), with 29 combinations tested across the experimental matrix [34].

#### 3.7.3. Comparison of Active Component Contents in ASC Stems and ASF

The optimized extraction process was applied to isolate active components from both ASF and ASC stems, followed by a comparative analysis of their content.

### 3.8. Evaluation of Active Components in Extracts from ASC Stems and ASF Stems

#### 3.8.1. Antibacterial Activity

The antibacterial activity of the extracts was evaluated using the paper disc diffusion method, where sterilized filter paper discs were utilized in petri dishes [35]. Four sample groups were tested: an antibiotic solution (Kanamycin) as a positive control (a), ethanol solution as a negative control(b), extract from ASC stems (c),and extract from ASF stems (d). Bacterial suspensions of *Pseudomonas aeruginosa*, *Staphylococcus aureus*, *Escherichia coli*, *Bacillus subtilis*, and *Candida albicans* were spread evenly onto culture media using the spread plate technique. The filter paper discs, soaked in their respective solutions, were placed on the bacteria-coated media and incubated at 37 °C for 24 h. The diameters of the resulting inhibition zones were measured, and each experiment was repeated in triplicate to obtain average inhibition zone diameters [36].

#### 3.8.2. Antioxidant Activity

##### DPPH Assay

The DPPH radical scavenging activity was assessed following a modified version of Sridhar’s method [37]. A solution (A) was prepared by dissolving 2.01 mg of DPPH in anhydrous ethanol to a final volume of 50 mL. Extracts from ASF and ASC stems were dissolved in anhydrous ethanol and diluted to create a concentration gradient (1.0, 0.8, 0.6, 0.4, 0.2, and 0.1 mg/mL), designated as solution B. Solutions A and B were mixed and incubated in the dark at room temperature for 30 min. The absorbance of the mixture was measured at 517 nm using a UV-visible spectrometer (UV-1600, Shanghai Mapada instrument Co., Ltd., Shanghai, China), with ascorbic acid (Vc) as the reference standard. The DPPH radical scavenging rate was calculated using Formula (13).

(13) $$\%Inhibition=\left[1-\frac{\left(A_{1}-A_{2}\right)}{A_{0}}\right]\times 100$$

In this formula, $A_{0}$ represents the absorbance of the blank solution, $A_{1}$ denotes the absorbance of the sample solution, and $A_{2}$ represents the background absorbance of the sample solution.

##### ABTS Assay

Following a modified version of Sridhar’s method [37], different concentrations of the samples (0, 0.8, 0.6, 0.4, 0.2, and 0.1 mg/mL) were prepared and added to the ABTS solution. The mixtures were then incubated at room temperature in the dark for 10 min. Post-incubation, the absorbance was recorded at 734 nm using a spectrophotometer (UV-1600, Shanghai Mapada instrument Co., Ltd., Shanghai, China). Ascorbic acid (Vc) served as the positive control. The scavenging activity percentage was calculated as % inhibition using the previously mentioned Equation (14).

(14) $$\%Inhibition=\left[\frac{\left(A_{0}-A_{1}\right)}{A_{0}}\right]\times 100$$

In this formula, $A_{0}$ represents the absorbance of the control solution, and $A_{1}$ denotes the absorbance of the sample solution.

### 3.9. Data Analysis

Statistical analyses were conducted using a one-way analysis of variance (ANOVA) followed by Tukey’s post-hoc test at a 95% confidence level in IBM SPSS Statistics 22. Regression analysis and response surface optimization were performed using Design Expert 13 software [38]. All measurements were conducted in triplicate and reported as mean ± standard deviation.

## 4. Conclusions

This study employed the ultrasound-assisted enzymatic extraction (UAEE) method to isolate active components from ASF stems, using the AHP-EWM method to weight the contents of key compounds—eleutheroside B, chlorogenic acid, eleutheroside E, hyperoside, and isofraxidin. The highest comprehensive score 94.76 was achieved under the optimal extraction conditions, that is an ultrasonic temperature of 59 °C, an ultrasound time of 57 min, an ethanol concentration of 61%, and a solid-to-liquid ratio of 39:1. The corresponding concentrations were: eleutheroside B (1.518 mg/mL), chlorogenic acid (0.600 mg/mL), eleutheroside E (1.192 mg/mL), isofraxidin (1.608 mg/mL), and hyperoside (0.270 mg/mL), representing a roughly 30% improvement in yield compared to UAE without enzymes. Further comparisons of the antibacterial and antioxidant activities of ASC and ASF stem extracts revealed that both exhibited significant antibacterial and antioxidant properties. Additionally, ASF stem extracts demonstrated superior antioxidant activity, which might be caused by the higher contents of hyperoside and eleutheroside B. These findings provide a scientific foundation for the potential substitution of ASC with ASF in future applications and broaden the application scope of ASF in fields such as pharmaceuticals, health supplements, and functional foods.

## Figures and Tables

**Table 1:** Factor levels used in the orthogonal experiments with different enzymes and dosages.

Levels	A Cellulase (%)	B Pectinase (%)	C Papain (%)
1	5	3	3
0	6	4	4
−1	7	5	5

**Table 2:** Orthogonal experimental results for the composite enzymes.

	Factor	Cellulase	Pectinase	Papain	Blank	Result
No.						
1		1	1	1	1	82.01
2		1	2	2	2	84.58
3		1	3	3	3	85.77
4		2	1	2	3	90.02
5		2	2	3	1	91.30
6		2	3	1	2	89.87
7		3	1	3	2	97.95
8		3	2	1	3	96.11
9		3	3	2	1	99.85
K1		252.36	269.98	267.99	273.15	
K2		271.19	271.98	274.45	272.40	
K3		293.91	275.49	275.02	271.91	
k1		84.12	89.99	89.33	91.05	
k2		90.40	90.66	91.48	90.80	
k3		97.97	91.83	91.67	90.64	
R		13.85	1.17	2.34	0.25	

**Table 3:** ANOVA of different enzymes and dosages.

Source	Sum of Squared Deviations	df	Mean Square	F-Value	*p*-Value	Significance
A	288.66	2	144.33	1106.07	0.001	**
B	5.19	2	2.59	19.88	0.048	*
C	10.16	2	5.08	38.93	0.025	*
Error	0.26	2	0.13			

Note: * denotes significance at *p* < 0.05, and ** denotes high significance at *p* < 0.01.

**Table 4:** Response surface experimental results for the UAEE of active components from ASF.

Run	Temperature (A)	Time (B)	Ethanol Concentration (C)	Solid-to-Liquid Ratio (D)	Comprehensive Score
1	50	80	60	50	79.02
2	50	60	60	40	98.26
3	50	40	70	40	81.88
4	40	60	50	40	80.23
5	50	60	70	30	82.57
6	50	60	50	50	80.49
7	50	80	70	40	79.64
8	50	80	50	40	81.75
9	60	80	60	40	80.51
10	50	60	70	50	81.20
11	60	60	60	50	83.64
12	50	60	60	40	97.59
13	50	40	50	40	81.99
14	40	60	60	50	75.02
15	50	40	60	50	76.74
16	50	60	50	30	85.13
17	50	80	60	30	78.69
18	50	60	60	40	97.76
19	60	40	60	40	86.01
20	50	40	60	30	83.25
21	40	40	60	40	73.30
22	60	60	50	40	85.02
23	60	60	60	30	85.68
24	50	60	60	40	97.58
25	50	60	60	40	98.24
26	40	60	70	40	76.20
27	40	80	60	40	76.12
28	60	60	70	40	87.54
29	40	60	60	30	77.51

**Table 5:** ANOVA results for the response surface analysis.

Source	Sum of Squares	df	Mean Squares	F-Value	*p*-Value	Significance
Model	1530.63	14	109.33	1184.20	<0.0001	***
A-temperature	208.58	1	208.58	2259.15	<0.0001	***
B-time	4.61	1	4.61	49.88	<0.0001	***
C-ethanol concentration	2.61	1	2.61	28.27	0.0001	**
D-solid-to-liquid ratio	23.26	1	23.26	251.94	<0.0001	***
AB	17.33	1	17.33	187.67	<0.0001	***
AC	10.75	1	10.75	116.39	<0.0001	***
AD	0.05	1	0.05	0.5581	0.4674	
BC	0.99	1	0.99	10.77	0.0055	**
BD	11.71	1	11.71	126.80	<0.0001	***
CD	2.69	1	2.69	29.10	<0.0001	***
A^2^	512.76	1	512.76	5553.87	<0.0001	***
B^2^	632.65	1	632.65	6852.44	<0.0001	***
C^2^	298.58	1	298.58	3234.05	<0.0001	***
D^2^	482.20	1	482.20	5222.88	<0.0001	***
Residual	1.29	14	0.09			
Lack of fit	0.84	10	0.08	0.73	0.69	
Pure error	0.46	4	0.11			
Cor total	1531.92	28				

Note: R^2^ = 0.9992, $R_{Adj}^{2}\;$ = 0.9983, $R_{Pre}^{2}\;$ = 0.9963, C.V.% = 0.3628. *** (*p* < 0.001), ** (*p* < 0.01).

**Table 6:** Criteria for the nine-level scale method.

Importance Score	Definition
1	Indicating that two factors are equally important
3	Indicating that one factor is slightly more important than the other
5	Indicating that one factor is clearly more important than the other
7	Indicating that one factor is strongly more important than the other
9	Indicating that one factor is extremely more important than the other
2, 4, 6, 8	The intermediate value between the two adjacent judgments above

**Table 7:** Pairwise comparison matrix of the main active ingredients in ASF stems.

Weight Index	Chlorogenic Acid	Eleutheroside B	Eleutheroside E	Hyperoside	Isofraxidin
Chlorogenic acid	1	1/7	1/7	1/5	3
Eleutheroside B	7	1	1	3	9
Eleutheroside E	7	1	1	3	9
Hyperoside	5	1/3	1/3	1	7
Isofraxidin	1/3	1/9	1/9	1/7	1

## Data Availability

Data are contained within the article.

## Supplementary Materials

The following supporting information can be downloaded at https://www.mdpi.com/article/10.3390/molecules30020397/s1: Figures S1–S3. Comparison of Active Component Contents in ASF Stems Extracted by UAE and UAEE; Comparison of active ingredient contents of ASC stems, and ASF stems under optimal extraction conditions; Comparison of the bacteriostatic effect of stem extracts of ASF and ASC on different strains of bacteria. Tables S1–S11. Diameter size of the circle of inhibition of stem extracts of ASF and ASC; Weighting factors for cellulase target compounds; Weighting factors for pectinase target compounds; Weighting factors for papain target compounds; Weighting coefficients of target compounds for orthogonal experiments; Weighting coefficients of target compounds for compound enzyme dosage; Weighting coefficients of target compounds for ultrasonic temperature; Weighting coefficients of target compounds for ultrasound time; Weighting coefficients of target compounds for liquid-to-material ratio; Weighting coefficients of target compounds for ethanol concentration; Weighting coefficients of target compounds in response surface experiments.
