# Supplementary material for: Ultrasound-Assisted Enzymatic Extraction of the Active Components from Acanthopanax sessiliflorus Stem and Bioactivity Comparison with Acanthopanax senticosus
Source: Molecules. 2025 Jan 18;30(2):397. doi: 10.3390/molecules30020397 (PMC11767547; doi:10.3390/molecules30020397)
Supplement: Supplementary file 1 [file molecules-30-00397-s001.zip › molecules-3382338-supplementary.pdf]

Supplementary information

Ultrasound-assisted enzymatic extraction of the active components from *Acanthopanax sessiliflorus* stem and bioactivity comparison with *Acanthopanax senticosus*

Qiaomu You, Xiongfei Luo*, Zhonghua Tang*

Key Laboratory of Forest Plant Ecology of Ministry of Education., Northeast Forestry University, Hexing Road 26, Harbin 150040, P.R. China.

E-mail address: lxf@nefu.edu.cn (X. Luo), tangzh@nefu.edu.cn (Z. Tang).

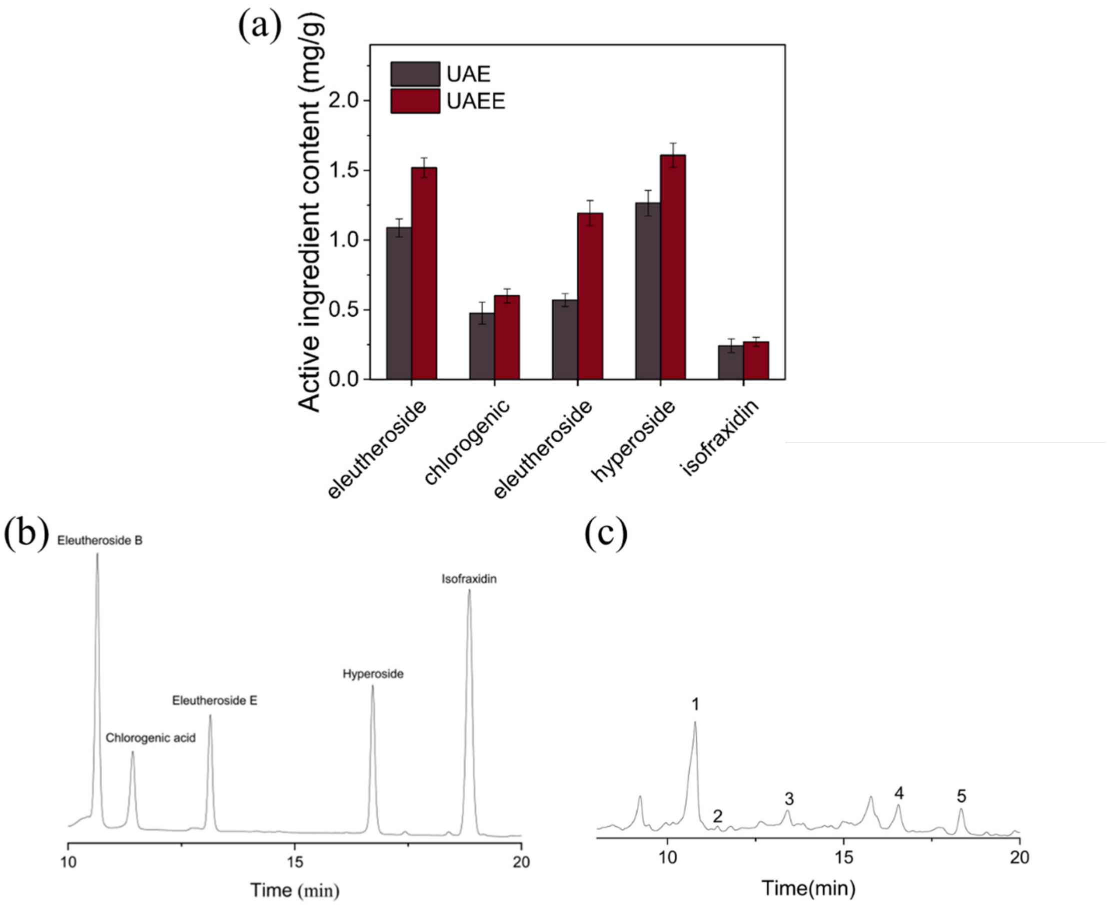

Figure S1. (a) Comparison of active component contents in ASF stems extracted by UAE and UAEE, HPLC chromatogram of standard substances (b) and identified compounds of extractions (c).

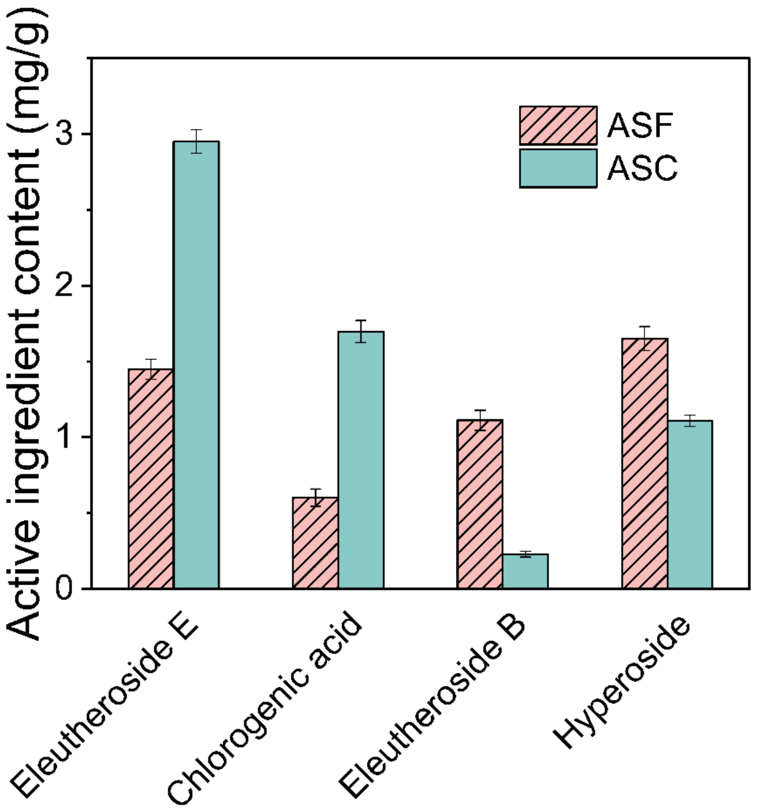

Figure S2. Comparison of active ingredient contents of ASC stems and ASF stems under optimal extraction conditions

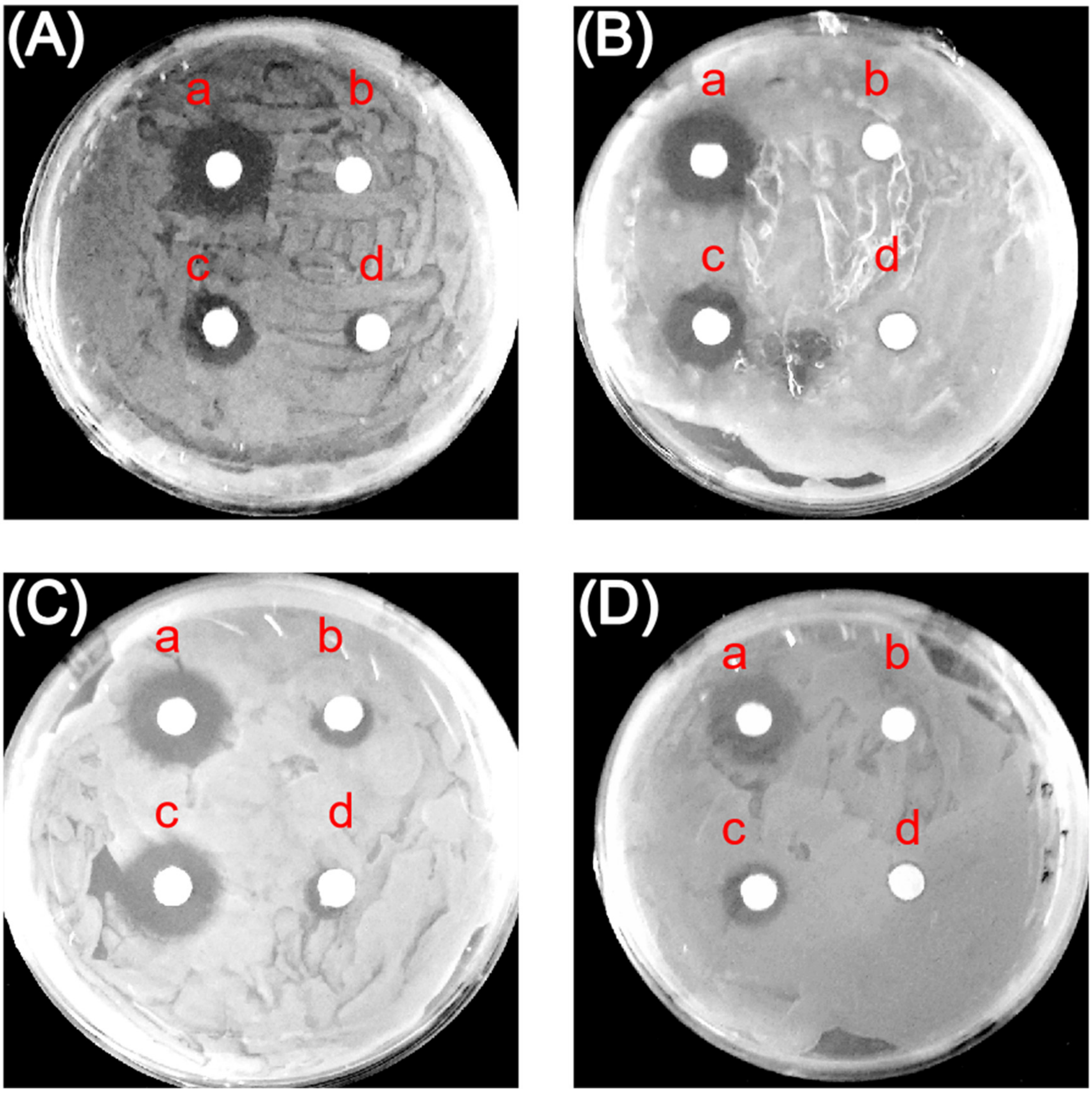

Figure S3. Comparison of the bacteriostatic effect of stem extracts of ASF and ASC on different strains of bacteria. Positive control (a), Negative control (b), ASC (c), ASF (d). *Pseudomonas aeruginosa* (A), *Bacillus subtilis* (B), *Candida albicans* (C), *Escherichia coli* (D).

Table S1 Diameter size of the circle of inhibition of stem extracts of ASF and ASC.

Sample	*Pseudomonas aeruginosa*	*Bacillus subtilis*	*Candida albicans*	*Escherichia coli*
antibiotic	11.30±1.28	8.85±0.59	10.60±0.78	8.14±1.64
53% ethanol solution	0.00±0.00	0.00±0.00	0.93±0.27	0.00±0.00
Stem Extract of AS	6.55±1.03	6.60±0.36	7.84±1.23	2.79±0.51
Stem Extract of ASS	2.53±0.47	0.19±0.12	2.95±0.53	0.11±0.02

Table S2 Weighting factors for cellulase target compounds

Methods	Weighting coefficients				
	Eleutheroside B	Chlorogenic acid	Eleutheroside E	Isofraxidin	Hyperoside
EWM	0.1463	0.3436	0.1831	0.1575	0.1695
AHP-EWM	0.3089	0.1148	0.3864	0.1595	0.0304

Table S3 Weighting factors for pectinase target compounds

Methods	Weighting coefficients				
	Eleutheroside B	Chlorogenic acid	Eleutheroside E	Isofraxidin	Hyperoside
EWM	0.1225	0.1946	0.1069	0.4673	0.1086
AHP-EWM	0.2481	0.0624	0.2166	0.4542	0.0187

Table S4 Weighting factors for papain target compounds

Methods	Weighting coefficients				
	Eleutheroside B	Chlorogenic acid	Eleutheroside E	Isofraxidin	Hyperoside
EWM	0.1378	0.2166	0.1124	0.2327	0.3005
AHP-EWM	0.3268	0.0813	0.2665	0.2649	0.0606

Table S5 Weighting coefficients of target compounds for orthogonal experiments

Methods	Weighting coefficients				
	Eleutheroside B	Chlorogenic acid	Eleutheroside E	Isofraxidin	Hyperoside
EWM	0.1992	0.1405	0.1654	0.2706	0.2243
AHP-EWM	0.3718	0.0415	0.3087	0.2424	0.0356

Table S6 Weighting coefficients of target compounds for compound enzyme dosage

Methods	Weighting coefficients				
	Eleutheroside B	Chlorogenic acid	Eleutheroside E	Isofraxidin	Hyperoside
EWM	0.2158	0.1437	0.2843	0.1405	0.2157
AHP-EWM	0.3546	0.0374	0.4672	0.1108	0.0301

Table S7 Weighting coefficients of target compounds for ultrasonic temperature

Methods	Weighting coefficients				
	Eleutheroside B	Chlorogenic acid	Eleutheroside E	Isofraxidin	Hyperoside

EWM	0.2255	0.2313	0.1249	0.1714	0.2469
AHP-EWM	0.4600	0.0746	0.2548	0.1677	0.0428

Table S8 Weighting coefficients of target compounds for ultrasound time

Methods	Weighting coefficients				
	Eleutheroside B	Chlorogenic acid	Eleutheroside E	Isofraxidin	Hyperoside
EWM	0.2525	0.2027	0.2685	0.1240	0.1523
AHP-EWM	0.4036	0.0513	0.4293	0.0951	0.0207

Table S9 Weighting coefficients of target compounds for liquid to material ratio

Methods	Weighting coefficients				
	Eleutheroside B	Chlorogenic acid	Eleutheroside E	Isofraxidin	Hyperoside
EWM	0.1667	0.1968	0.2158	0.1663	0.2544
AHP-EWM	0.3237	0.0604	0.4189	0.1549	0.0420

Table S10 Weighting coefficients of target compounds for ethanol concentration

Methods	Weighting coefficients				
	Eleutheroside B	Chlorogenic acid	Eleutheroside E	Isofraxidin	Hyperoside
EWM	0.1844	0.2652	0.2042	0.1743	0.1718
AHP-EWM	0.3487	0.0793	0.3862	0.1582	0.0276

Table S11 Weighting coefficients of target compounds in response surface experiments

Methods	Weighting coefficients				
	Eleutheroside B	Chlorogenic acid	Eleutheroside E	Isofraxidin	Hyperoside
EWM	0.1415	0.2409	0.2979	0.1843	0.1415
AHP-EWM	0.2450	0.0660	0.5159	0.1531	0.2450
